# A new species of *Hyperimerus* Girault from China, with a key to species of the genus (Hymenoptera, Chalcidoidea, Pteromalidae)

**DOI:** 10.3897/zookeys.817.29886

**Published:** 2019-01-15

**Authors:** Lan Yang, Da-wei Huang, Hui Xiao

**Affiliations:** 1 Key Laboratory of Zoological Systematics and Evolution, Institute of Zoology, Chinese Academy of Sciences, Beijing, 100101, China Hebei University Baoding China; 2 College of Life Science and Technology, Hebei University, Baoding, 071002, China Institute of Zoology, Chinese Academy of Sciences Beijing China

**Keywords:** China mainland, key, *
Hyperimerus
*, new species, Pteromalidae, taxonomy

## Abstract

A new species of *Hyperimerus* Girault (Hymenoptera: Pteromalidae), *H.sichuanicus* Yang & Xiao, **sp. n.**, is reported and described for the first time from mainland China. A key to the worldwide species of *Hyperimerus* and illustrations of external features of the species are also provided.

## Introduction

The genus *Hyperimerus* was erected by Girault (1917) to include *Hyperimeruscorvus* Girault from California, USA, and the genus was placed in the subfamily Erimerinae of Callimomidae. Subsequently, *Hyperimerus* was put in the tribe Asaphini under Pteromalidae by Peck (1951). Graham (1969) upgraded the tribe Asaphini to subfamily Asaphinae. Since then, several researchers reported *Hyperimerus* from Europe (Graham 1969; Bouček 1977; Dzhanokmen 1978; Kalina 1989; Ulrich 1999). Schender et al. (2014) reviewed the genus and redescribed the two species, *H.corvus* Girault and *H.pusillus* (Walker). Until now, two valid species of *Hyperimerus* are known in the world. Only one species, *H.pusillus* (Walker), has previously been reported in China (Huang and Xiao 2005). In this study, one new species, *H.sichuanicus* Yang & Xiao, sp. n., is described.

## Materials and methods

All specimens for the present study were swept using an insect net and preserved in 95% ethanol. They were subsequently air dried, point-mounted and examined with a LEICA MZ APO stereomicroscope. Photographs were taken under the Nikon Multizoom AZ100 system, and the plates were compiled using Adobe Photoshop software. In addition, the author also examined the specimens of *Hyperimerus* deposited in the National History Museum, London in 2002. All type specimens of the new species are deposited in the Institute of Zoology, Chinese Academy of Sciences, China (IZCAS).

Morphological terminology mostly follows that of Bouček (1988) and Gibson et al. (1997). All specimens were examined and identified based on the studies of Graham (1969), Gibson and Vikberg (1998), Bouček and Rasplus (1991) and Schender et al. (2014). The new species is described based on the holotype specimen. Body length excluding the ovipositor sheaths is measured in millimetres (mm); other measurements are given as ratios. Abbreviations of morphological terms used are:

**Fu_n_** funicular segment number;

**POL** posterior ocellar distance;

**OOL** ocellocular distance;

**Gt_n_** gastral tergum number.

## Taxonomy

### Key to species

**Table:** 

1	Fu_1_ shorter than pedicel; ovipositor sheaths shortly protruded, shorter than 1/2 length of gaster	**2**
–	Fu_1_ longer than pedicel; ovipositor sheaths distinctly protruded, ca. 2/3 length of gaster	***H.corvus* Girault**
2	Clava as long as Fu_5_-Fu_7_ combined; marginal fringe of outer margin of fore wing longer than length of uncus; propodeum with irregularly and densely areolate sculptures (Fig. 5), most cells of sculpture as big as propodeal spiracles	***H.sichuanicus* sp. n.**
–	Clava shorter than length of Fu_5_-Fu_7_ combined; marginal fringe of outer margin of fore wing shorter than length of uncus; propodeum with irregularly and sparsely sculptures (Fig. 10), most cells of sculpture more than 2× as big as propodeal spiracles	***H.pusillus* (Walker)**

#### Hyperimerus

Taxon classificationAnimaliaHymenopteraPteromalidae

Girault, 1917

Hyperimerus Girault, 1917: 5. Type species: Hyperimeruscorvus Girault, by original designation.
Hyperimerus Girault: Graham 1969: 83–84; Huang and Xiao 2005: 281–282; Schender et al. 2014: 408–420.
Mespilon Graham, 1957: 406. Type species: Mespilonexiguum Graham, by original designation. Synonymized by Bouček 1965: 549.

##### Diagnosis.

Body with dense hairy, head and mesosoma with engraved reticulate sculpture. Head subtriangular in frontal view; antennal scrobes deep, not reaching anterior ocellus; malar sulci distinct; antennal insertion obviously below centre of face; formula 11173. Head in dorsal view, occiput with horseshoe-like carina. Pronotum quadrangular; notauli complete, frenal line on scutellum indistinct; propodeal sculptures irregular; fore wing with pilosity, speculum absent; postmarginal vein longer than marginal vein. Gaster convex, petiole transverse, Gt_1_ and Gt_2_ large and smooth, ovipositor sheath exerted.

*Hyperimerus* is similar to *Asaphes* in the subfamily Asaphinae, but it can be recognized by the antenna with one anellus and seven funicular segments (two anelli and six funicular segments in *Asaphes*), frenal line on scutellum indistinct (distinct in *Asaphes*), petiole transverse (petiole longer than broad and with longitudinal ridges in *Asaphes*).

##### Biology.

*Hyperimerus* is recorded as parasites of *Pseudococcus* (Hemiptera: Pseudococcidae), *Psylla* (Hemiptera: Psyllidae), *Lymantria* (Lepidoptera: Erebidae), *Choristoneura*, *Zeiraphera* (Lepidoptera: Tortricidae), *Chrysopa* (Neurop: Chrysopidae), *Hemerobius*, and *Sympherobius* (Neuroptera: Hemerobiidae) (Graham 1969; Burks 1979; Schender et al. 2014; Noyes 2018). The genus is associated with the following plants: *Fagussylvatica*, *Pyruscommunis* (Dzhanokmen 1978; Ghahari et al, 2010).

##### Distribution.

China (Sichuan, Tibet); Holarctic, Oriental, and Neotropical regions.

#### Hyperimerus sichuanicus

Taxon classificationAnimaliaHymenopteraPteromalidae

Yang & Xiao sp. n.

http://zoobank.org/36329DB9-9E34-44E8-8CEB-D7773E07A6AA

##### Diagnosis.

Antenna with Fu_1_ shorter than pedicel; Fu_7_ quadrate, clava as long as Fu_5_-Fu_7_ combined; marginal fringe of outer margin of fore wing longer than length uncus; postmarginal vein 1.65× as long as stigmal vein; propodeum with irregularly and densely sculptured, most of them as big as propodeal spiracles; ovipositor sheaths protruded, not longer than 1/2 length of gaster.

##### Description.

*Female* (holotype). Body length 1.4 mm. Head and body dark green with bluish reflection. Mandibles brown, antennae dark brown. Coxae concolorous with body, remainder of legs brown except tibiae yellowish brown, colour gradation from yellowish brown to brown at tarsi.

**Figures 1–6.**
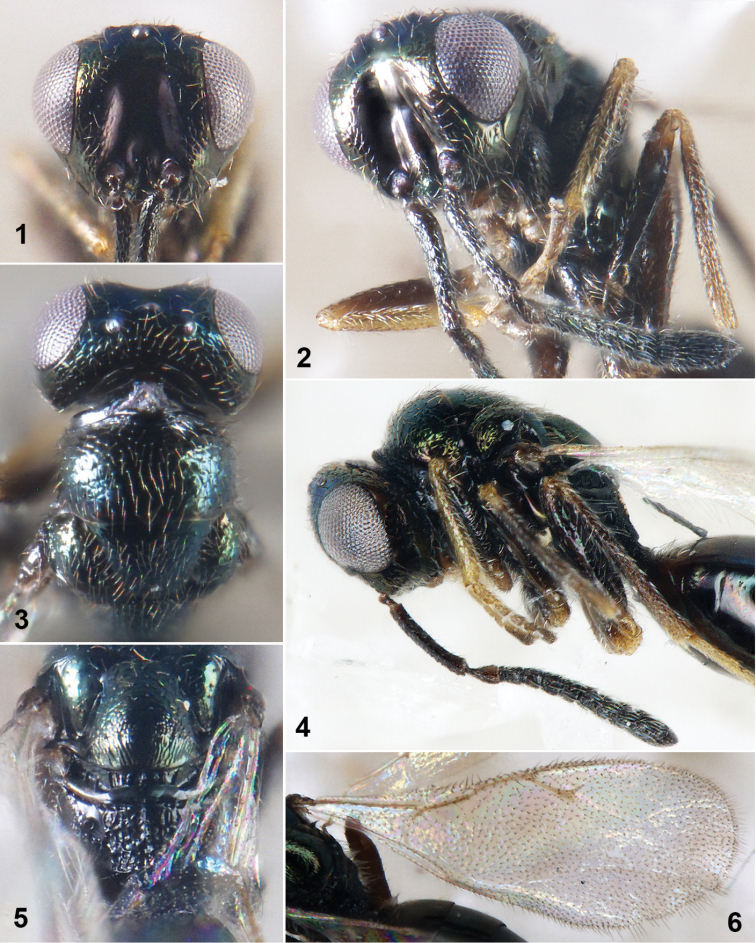
*Hyperimerussichuanicus* sp. n., female holotype **1** Head in frontal view **2** Head in lateral view **3** Head and pronotum in dorsal view **4** Antenna and thorax in lateral view **5** Propodeum in dorsal view **6** Fore wing in dorsal view.

Head in frontal view 1.26× as wide as high (Fig. 1); eyes height 0.62× head height; eyes separated by 1.24× their height. Antennal scrobes deep and glabrous, not reaching anterior ocellus (Fig. 2), interantennal crest distinct. Clypeal suture obvious, clypeus and supraclypeal area slightly prominent, clypeal margin truncate. Antennal insertion on lower ocular line, close to clypeal margin, distance from upper margin of torulus to lower margin of anterior ocellus 7× distance from lower margin of torulus to lower margin of clypeus. Gena smooth and convex; malar sulci distinct, malar space 0.67× eyes height. Antenna (Fig. 4) clavate; scape 1.14× eyes height, reaching anterior ocellus; length of pedicel and flagellum combined longer than head width (1.16×); pedicel conical, 2× as long as broad in lateral view; anellus transverse; each funicular segment and clava segment with single row of sensilla; ratio of length and width from Fu_1_ to Fu_7_ as 3:3, 4:3.5, 4:4, 5:4, 5:4, 5:5, 5:5; clava equal to length of Fu_5_-Fu_7_ combined. Head in dorsal view, 1.95× as wide as long (Fig. 3); vertex convex, POL 2.75× OOL.

Head 1.27× as broad as mesosoma. Mesosoma convex, 1.76× as long as broad. Pronotum, mesoscutum and anterior area of scutellum with engraved reticulation. Pronotum 0.91× as broad as mesoscutum. Mesoscutum 2× as broad as long; notauli deep and complete. Scutellum weakly convex, frenal line obscured medially, frenum with longitudinal rugae (Fig. 5), frenum ca. 1/5 length of scutellum. Dorsellum glabrous, 4× as broad as long, 0.17× as long as propodeum. Propodeum (Fig. 5) 0.71× as long as scutellum, median carina distinct; areolate sculptures on median area irregular and dense, most cells as big as propodeal spiracles; area otherwise glabrous; propodeal spiracles ovate, separated from metanotum by 1.5× spiracular width; callus densely hairy laterally and posteriorly. Prepectus smooth and with sparse hairy. Upper mesepisternum setose and finely reticulate, area otherwise smooth. Fore wing (Fig. 6) 2.63× as long as broad, speculum absent; marginal fringe of outer margin of fore wing longer than length of uncus; costal cell with sparse setae; ratio of submarginal vein: marginal vein: postmarginal vein: stigmal vein as 49:18:26:16; stigmal vein oblique, stigma slightly expand, uncus ca. 1/4 length of stigmal vein.

Gaster ovate, 1.88× as long as broad, shorter than head and mesosoma combined. Petiole short, 0.5× as long as broad, dorsum with longitudinal ridges. Each gastral tergite smooth; Gt_1_ with long hairy around petiole; Gt_1_ and Gt_2_ large and smooth, 0.47× length of gaster. Ovipositor sheaths distinctly produced in dorsal view; gaster in lateral view, ovipositor sheaths 0.47× length of gaster, 0.76× length of hind tibia.

Male. Body slender, scape yellowish-brown, antenna segmented clearly, others similar to the female.

##### Remarks.

The new species is similar to *H.corvus*, but different by the antenna with Fu_1_ shorter than pedicel, ovipositor sheaths shortly protruded. It is also very close to *H.pusillus* in having the Fu_1_ shorter than pedicel, ovipositor sheaths shorter than 1/2 length of gaster, but can be recognized by the characters listed in the key.

##### Material examined.

Holotype. China, ♀, Sichuan: Kangding, 30.04°N, 101.57°E, 15.VI.2017, leg. Yanzhou Zhang (Hyp-2018-06, original number ZYZ-2017-28). Paratypes. 1♀1♂, Sichuan: Kangding, 29.VI.2017, leg. Yanzhou Zhang (Hyp-2018-01, Hyp-2018-02, original number ZYZ-2017-08); 1♀, Sichuan: Kangding, 2.VIII.2017, leg. Yanzhou Zhang (Hyp-2018-03, original number ZYZ-2017-20); 1♂, Sichuan: Kangding, 29.VI.2017, leg. Yanzhou Zhang (Hyp-2018-08, original number ZYZ-2017-029).

##### Etymology.

Named after the location of the type material.

##### Hosts.

Unknown.

##### Distribution.

China (Sichuan).

#### Hyperimerus pusillus

Taxon classificationAnimaliaHymenopteraPteromalidae

(Walker, 1833)

Cyrtogaster pusilla Walker, 1833: 383. Holotype female (BMNH No. 3300), examined.
Hyperimerus pusillus (Walker): Graham 1969: 83; Bouček 1993: 1306; Huang and Xiao 2005: 281–282; Schender et al. 2014: 414–417.
Mespilon exiguum Graham, 1957: 406. Synonymized by Graham 1969: 83.

##### Diagnosis.

**Figures 7–10.**
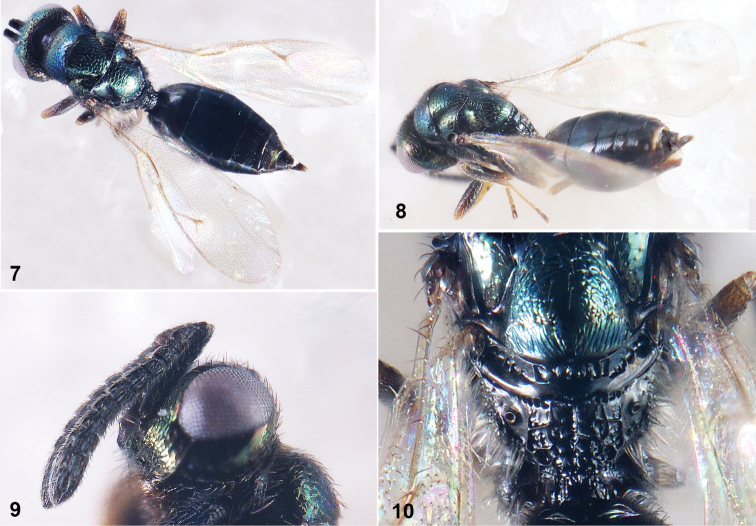
*Hyperimeruspusillus* (Walker, 1833) **7** Body in dorsal view **8** Body in lateral view **9** Head and antenna in lateral view **10** Propodeum in dorsal view.

Female. Body (Fig. 7, 8) length approximately 1.5 mm, dark green. Head width 1.32× height in frontal view. Eyes height 0.58× head height; eyes separated by 1.07× their height. Gena with malar sulci distinct, malar space 0.55× eyes height. Antenna (Fig. 9) with scape reaching anterior ocellus; pedicel and flagellum combined 1.2× head width; pedicel broader than Fu_1_, clava shorter than length of Fu_5_ – Fu_7_ combined. Scutellum with frenum ca. 1/3 length of scutellum. Propodeum with areolate sculptures irregular and sparse, most of sculptures 2× as big as propodeal spiracles (Fig. 10). Marginal fringe of outer margin of fore wing shorter than length of uncus; marginal vein shorter than postmarginal vein (0.59×), postmarginal vein approximately 1.85× as long as stigmal vein. Petiole 0.4× as long as broad. Gaster 1.91× as long as broad; narrower than thorax, shorter than the length of head and mesosoma combined. Ovipositor sheaths slightly produced in dorsal view; gaster in lateral view, ovipositor sheaths 0.26× length of gaster, 0.44× length of hind tibia.

##### Material examined.

China, 1♀2♂, Sichuan: Kangding, 15.VI.2017, leg. Yanzhou Zhang (Hyp-2018-07, original number ZYZ-2017-28; Hyp-2018-09, original number ZYZ-2017-29); 1♀, Sichuan: Kangding, 2.VIII. 2017, leg. Yanzhou Zhang (Hyp-2018-04, original number ZYZ-2017-21); 1♀, Sichuan: Kangding, 29.VI.2017, leg. Yanzhou Zhang (Hyp-2018-11, original number ZYZ-2017-42); 1♂, Tibet: Zhamo, 2700m, VII. 19.1978, leg. Fasheng Li.

##### Hosts.

Parasitoids of *Psyllapyricola* (Ghahari et al., 2010), *Phenacoccusaceris*, *Psyllaulmi* (Dzhanokmen, 1978).

##### Distribution.

China (Sichuan, Tibet), Holarctic, Oriental, and Neotropical regions.

## Supplementary Material

XML Treatment for Hyperimerus

XML Treatment for Hyperimerus sichuanicus

XML Treatment for Hyperimerus pusillus
